# A concern over terminology in vaccine effectiveness studies

**DOI:** 10.2807/1560-7917.ES.2018.23.10.18-00103

**Published:** 2018-03-08

**Authors:** Benjamin J Cowling, Sheena G Sullivan

**Affiliations:** 1 WHO Collaborating Centre for Infectious Disease Epidemiology and Control, School of Public Health, The University of Hong Kong, Hong Kong Special Administrative Region, China; 2 WHO Collaborating Centre for Reference and Research on Influenza at the Peter Doherty Institute for Infection and Immunity, Melbourne, Victoria, Australia; 3 Department of Epidemiology, Fielding School of Public Health, University of California, Los Angeles, Los Angeles, California; 4 Centre for Epidemiology and Biostatistics, Melbourne School of Population and Global Health, University of Melbourne, Melbourne, Victoria, Australia

**Keywords:** viral infections, influenza, influenza virus, vaccines and immunisation

**To the editor:** Ongoing systematic monitoring of vaccine effectiveness (VE) provides evidence to support vaccination programmes and policies. A series of recent articles in *Eurosurveillance* [1-4] and elsewhere [5], continue to provide timely estimates of influenza VE from around the world. These reports are useful for supporting public health decision making on the use of influenza vaccines, which are the best means currently available for reducing the considerable burden of influenza.

However, as we pointed out already, we are concerned that the continued use of the terms ‘crude VE’ and ‘adjusted VE’ in many such papers is unhelpful [6]. The term vaccine *effectiveness* implies an attempt to measure a causal estimate, i.e. the *effect* of vaccination on the risk of an infection-related outcome such as medically-attended influenza or hospitalisation, and not merely the association of vaccination and (absence of) influenza virus infection [6]. The term ‘effect’ should consequently be reserved for the reporting of unbiased estimates of a causal effect, or at least the reasonable attempt to generate such an unbiased estimate.

Epidemiologists have long been cautioned against drawing causal inferences from observational studies [7]. Indeed, some specialist epidemiology journals discourage use of the word ‘effect’. We are instead encouraged to comment on whether a particular factor is ‘associated with reduced risk of…’ rather than stating definitively that it ‘reduced the risk of…’ [8]. However it is increasingly realised that observational studies can, in certain cases, permit inferences on cause and effect relationships [9,10].

In a test-negative design study of influenza vaccine effectiveness against medically-attended influenza, a crude association between case vs control status and influenza vaccination history may not reflect the true strength of a causal effect. The association may be confounded by a factor such as age, i.e. a factor that has a causal effect on both the exposure (vaccination) and the outcome (influenza virus infection). An estimate of the *effect* of vaccination on risk of medically-attended influenza would need to take into account any potential confounding by age or other factors, which may be achieved by methods such as stratification or regression analysis. Typically, the VE estimate would be derived from the antilog of the estimated coefficient for vaccination in a regression model that included potential confounders; this value is often referred to as the adjusted odds ratio (AOR). In the special case where all potential confounders, but no other variables, are included as covariates in the regression model, and in the absence of other biases [6], it is possible to interpret the AOR as an estimate of a causal effect, and estimate the VE as one minus the AOR.

In contrast, crude (i.e. unadjusted) estimates are unlikely to be an accurate estimate of the VE, because of confounding. Discussion of crude associations should therefore remain on the odds ratio scale to prevent the reader assuming they are a measure of effect. The causal effect is not the quantity that has been adjusted for confounding, it is based on an estimate from an analysis that accounts for confounding. We therefore recommend avoiding the terms ‘crude VE’ and ‘adjusted VE’. In summary tables, it is unnecessary to report unadjusted odds ratios or ‘crude VE’. If authors wish to compare unadjusted and adjusted odds ratios they could be presented separately, for example in an appendix.
